# Mechanism Analysis of the Inverse Doppler Effect in Two-Dimensional Photonic Crystal based on Phase Evolution

**DOI:** 10.1038/srep24790

**Published:** 2016-04-22

**Authors:** Qiang Jiang, Jiabi Chen, Yan Wang, Binming Liang, Jinbing Hu, Songlin Zhuang

**Affiliations:** 1 Shanghai Key Lab of Contemporary Optical System, Optical Electronic Information and Computer Engineering College, University of Shanghai for Science and Technology, Shanghai 200093 China; 2 College of Physics and Communication Electronics, Jiangxi Normal University, Nanchang 330022 China

## Abstract

Although the inverse Doppler effect has been observed experimentally at optical frequencies in photonic crystal with negative effective refractive index, its explanation is based on phenomenological theory rather than a strict theory. Elucidating the physical mechanism underlying the inverse Doppler shift is necessary. In this article, the primary electrical field component in the photonic crystal that leads to negative refraction was extracted, and the phase evolution of the entire process when light travels through a moving photonic crystal was investigated using static and dynamic finite different time domain methods. The analysis demonstrates the validity of the use of *n*_*p*_ (the effective refractive index of the photonic crystal in the light path) in these calculations, and reveals the origin of the inverse Doppler effect in photonic crystals.

The inverse Doppler effect where an observer measures a higher frequency from a source moving away, is such an intriguing phenomenon that it has attracted substantial theoretical and experimental attention since it was first proposed^1^,^2^,^3^,^4^,^5^. Our team observed the inverse Doppler effect at optical frequencies for the first time in 2011^6^ using left-hand photonic crystal (LH-PhC)^7^. However, in that article, the equation


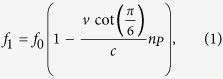


where *c* is the vacuum velocity of light, *f*_*0*_ is the original frequency of the light source (for instance, CO_2_ laser in ref. 6) and *f*_*1*_ is the Doppler frequency at the second interface of the PhC, was used to calculate the frequency shift, and was based on phenomenological theory. In that research, we considered the contribution of the LH-PhC by treating it as a homogenous medium with a negative *n*_*p*_ refractive index, and used the classic Doppler formula of a mechanical wave^8^,^9^,^10^, which makes no sense with *n*_*p*_ < 0. Hence, we were inspired to elucidate the physical mechanism underlying the inverse Doppler Effect in PhC and determine the rationale for using equation (1) to obtain the inverse frequency shift^6^.

We first address the controversy surrounding the explanation of the inverse Doppler effect in magnetic, nonlinear transmission lines^2^,^11^. N. Seddon and T. Bearpark attribute it to *v*_*g*_ · *v*_*p*_ < 0 (*v*_*g*_ is the group velocity and *v*_*p*_ is the phase velocity) in the second Brillouin zone (BZ), whereas Evan J. Reed considered a time-dependent reflection coefficient in the first BZ, with *v*_*g*_ · *v*_*p*_ > 0^12^,^13^. By contrast, Alexander Kozyrev demonstrated that the shift arises from the complex spatial structure of propagating waves, some of which have *v*_*g*_ · *v*_*p*_ < 0 resulting in the inverse Doppler Effect^14^. However, for light traveling through a LH-PhC, controversy exists regarding whether *v*_*p*_ is opposite to the wave vector *k* along the propagation direction^15^,^16^. Based on observations of Bloch harmonics and negative phase velocities in PhC waveguides resulting from the typical dispersion^17^, we believe that some spatial harmonics in LH-PhC have *v*_*p*_*·k* < 0 in terms of the decomposition of Bloch waves, which leads to the inverse Doppler effect in moving PhC.

In this article, we investigate the main spatial harmonics components of the complex field distribution in a LH-PhC and describe out the backward-propagating wave. Subsequently, by analyzing the phase evolution of the entire optical path using the static finite different time domain (FDTD) method, we obtain a Doppler shift that is in accordance with the experimental results. Finally, an improved dynamic FDTD method is proposed to reproduce the continuous motion of the PhC in the experiment^6^ to the greatest extent, and the rationality of *n*_*p*_, which was used in Eq. (1)^6^ is verified.

## Results

### Analysis of experimental setup and corresponding model

**Figure 1:**
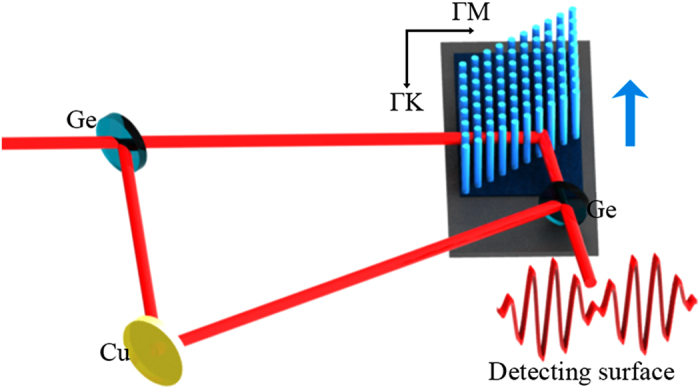
Schematic representation of the experimental setup used in ref.^6^. The blue arrow points in the direction of the moving platform, which holds the PhC and splitter.

To start our computation, first we analyze the experimental setup used in ref. 6. As it is shown in Fig. 1(a), a transverse magnetic (TM) polarized laser beam at the desired wavelength (*λ* = 10.6 μm) is divided into two beams by the first Ge splitter. The transmitted beam travels through the LH-PhC which is fixed on a moving platform, and the reflected beam is then reflected by a Cu reflector and the second Ge splitters, one of which is fixed on the moving platform. Finally, the two beams interfere on the detecting surface. The LH-PhC under investigation, which was achieved by electron beam Lithography, is a triangular silicon rod array prism with a vertex angle of rhombus geometry of 60°, its effective refractive index (*n*_*eff*_) was measured to be −0.5062^6^. The radius of the silicon rods was estimated to be 0.226*a* (where *a* is the lattice constant, which was designed to be as 5 μm) from the equi-frequency surface (EFS), which is close to the design requirement^6^.

### Phase evolution of light traveling through PhC

**Figure 2:**
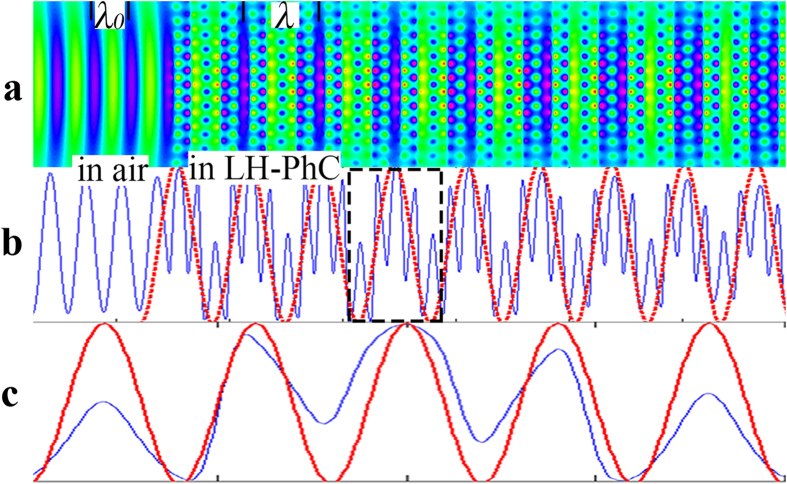
Component analysis of the field when light traveling though the LH-PhC. (**a**) Field distributions along the propagation direction in air and in the PhC. (**b**) The Original averaged field is labeled with a blue line, and the red line represents the spatial waveform of the electrical field component E_1_ extracted from the original field. (**c**). The blue line is a close look into the blue line in the dash rectangle of (**b**), and the red line indicates the extracted spatial waveform of the field component E_2_.

**Figure 3:**
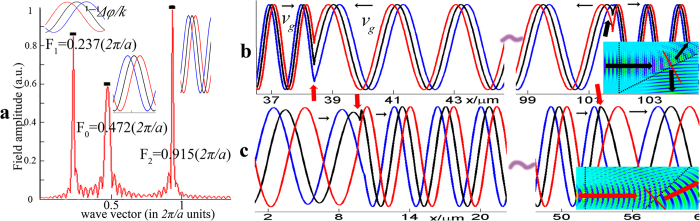
Phase changes for the main components along the propagation direction. (**a**) The spatial Fourier spectrum of the original field data reveals several spatial frequencies: F_0_, F_2_, and F_3_. The insets present the electrical field distributions of the incident wave, E_1_ and E_2_, at adjacent times, corresponding to F_0_, F_1_ and F_2_, the black lines shos field distribution at one moment, the blue lines shows the field distribution for the previous time, and the red lines shows the field distribution for the later time. (**b,c**) Spatial field distributions recorded along the light path as light passes through the LH-PhC and RH-PhC, The red arrows indicate the interfaces between the PhC and air. In the LH-PhC, the phase evolutions on either side of the interfaces are opposite. In the RH-PhC, the phase evolutions on either side are the same, and the insets show light propagating through the LH- PhC and RH- PhC. The black dotted lines denote the interface between the PhC and air.

To analyze the phase evolution in the LH-PhC as light passes through it, we employed the FDTD method to simulate the electrical field distribution and set the spacing grid to *a*/40 to ensure accuracy. We recorded the 2-D electrical field data (the size of the recorded data is 2000*7000) of light traveling in the LH-PhC and showed the corresponding spatial field distribution in Fig. 2(a). The entire electrical field distribution of light traveling through the LH-PhC and air is manifested in the inset of Fig. 3(b). In Fig. 2(a), the field distribution was periodic in both the PhC and air along the propagation direction. The periods were designated as *λ* and *λ*_*0*_, and the horizontal coordinate is the position along the propagation direction. We averaged the recorded data along the ΓK direction (perpendicular to the propagation direction), and obtained a 1*7000 matrix: this intensity-averaged field is shown in Fig. 2(b) as a blue-labeled line with periodic peaks that can be clearly observed.

The electrical field in a PhC can be decomposed into a series of plane waves with different amplitudes and frequencies because the field obeys the Bloch theorem. The Fast Fourier Transform (FFT) method is highly advantageous for analyzing the complex frequencies in a 2-D PhC^16^,^17^,^18^,^19^. Here, a 1-D FFT was applied to the averaged electrical field data, and the spectra are depicted in Fig. 3(a). We denote F_0_, F_1_, and F_2_ for the three peaks, and list their corresponding spatial frequencies in the form of wave vectors in Fig. 3(a). As expected, one of the spatial frequencies, 0.472(*2π/a*), is exactly the same as the actual spatial frequency of a CO_2_ laser in air as calculated from F_0_ = *a/λ*_*0*_ (*λ*_*0*_ = 10.6 μm). Other two peaks labeled with F_1_ and F_2_ are the spectra in the PhC. These two main components have large amplitudes, while there are also few tiny noises with small amplitude, which are arising from the numerical FFT. Differing with that in the homogenous left hand materials (LHMs), the beam propagation in the PhC takes the form of a Bloch wave, which can be decomposed into a series of complex plane wave; however, it is difficult to physically assign global phase-front velocities and single wave vector to the global Bloch wave. To overcome this issue, B. Lombardet proposed an appropriate way, which is to use the component of which the wave vector dominates the Fourier decomposition of Bloch wave to analyze the Bloch wave^19^. In Fig. 3(a), we can see that F_1_ and F_2_ possess most of the components’ energy, and thus, we focus on these two components in the following analysis.

For further study, we extracted the two main electrical field components E_1_ and E_2_ from the field in the PhC. More specifically, we filtered F_1_ and F_2_ from the spectrum and then applied an inverse fast Fourier Transform (iFFT). The corresponding reconstructed fields were shown in Fig. 2(b,c) with red lines. E_2_ is a close look into the dashed rectangle of E_1_, and E_1_ is in a manner similar to an envelope of E_2_ by modulating its amplitude.

Hence, we can assign two different refractive indexes for these two components when they propagate in the LH-PhC because of the unusual dispersion character of the LH-PhC, and the refractive index can be calculated as |*n*_*eff*_| = *λ*_*0*_*/λ*_*i*_ = F_i_/F_0_ (*i* = 1, 2), with two results of 0.5016 for E_1_ and 1.9406 for E_2_. Notably, the value 0.5016 is close to the absolute value of the measured value of the negative refraction index, 0.5062. If F_1_ corresponds to negative refraction, the sign for *n*_*eff*_ should be negative, which implied the component E_1_ propagates backward. E_2_ should be propagating forward. To confirm our speculation, we analyzed the phase evolution Δφ^20^ of the electrical field by recording the electrical field at adjacent time slots with an interval ΔT of 4.067*fs*, which is smaller than the light period, T = 35.3*fs*, to ensure the validity of the inferred propagation direction. The field distributions of the incident wave, components E_1_ and E_2_ are illustrated in the insets of Fig. 3(a), beside three peaks. Here, black lines represent the field distribution at one moment, blue lines represent the field distribution at the previous time, and the red lines represent the field distribution at the next time. As expected, for the incident wave and E_2_, the red line always leads the black line, indicating a phase lead, Δ*φ* > 0. By contrast, for E_1_, Δ*φ* is negative, suggesting a phase lag^21^. Considering the phase velocity *v*_*p*_ = Δ*φ*/(*k *· Δ*T*), for the incident wave and E_2_, *v*_*p*_ > 0, which means that the wave moves forward, *n*_*eff*_ = 1.9406 for E_2_. However, as for E_1_, *v*_*p*_ < 0, then *v*_*p*_ · *k* < 0, i.e., the phase velocity and the wave vector are antiparallel. Therefore, the phase of E_1_ is delayed, and *n*_*eff*_ is −0.5016 instead of 0.5016. E_1_ and E_2_ correspond to plane waves propagating with opposite phase velocities but they are coupled because of the periodicity and travel forward at the same group velocity.

As for E_1_, the corresponding wave vector is 0.237(2π/*a*), which is in the first BZ, because |*n*_*eff*_| = 0.5016 is smaller than that of air (*n*_*air*_ = 1), E_1_ can exit the interface of the PhC. Meanwhile, each point on the equi-phase surface of E_1_ undergoes different phase reduction when it exits obliquely from the interface of the LH-PhC to the normal medium. Thereby, under the phase conservation law, these points undergo different optical path and form a new equi-phase surface which is on the same side of normal as the incident beam, yielding the negative refraction phenomenon. However, for component E_2_, the corresponding wave vector is 0.915(*2π/a*), which is out of the first BZ, *n*_*eff*_ = 1.9406 is larger than n_air_, and because the critical angle (31°) is smaller than the incident angle (60°), total reflection occurs, thus it cannot couple to free-space, as illustrated by the black arrow in the inset of Fig. 3(b).

### Doppler shift obtained using the static FDTD method

The spatial distributions of the field inside and outside of the PhC were independently discussed above. Now let us consider the entire process of the transmission of light through a stationary PhC after the field stabilizes. Since E_1_ is the only component that can couple into free-space from the LH-PhC in an incident angle of 60° at the second interface of the LH-PhC, we extracted E_1_ from the recorded data of the PhC’s area at one time and treated it as the only contributing field in the PhC because *v*_*p*_ < 0. Then, we expanded the spatial field distributions in front of, inside and behind the PhC in the same optical path. Using the same process, the spatial field distributions at the previous and later times were all analyzed, as depicted in Fig. 3(b). The phase velocity directions are opposite on either sides of the interface between the air and PhC, which echoes the analysis above. For comparison, we designed an RH-PhC with a *n*_*eff*_ of 1.12 at the incident wavelength and *a* = 5 μm. At an incident wavelength of 31.25 μm, the corresponding propagation schedule inserted in Fig. 3(c) exhibited a normal refraction behavior. By performing the same analysis, the spatial field distribution was obtained, as shown in Fig. 3(c). Under these conditions, the phase change remained positive in both the air and the PhC, because the red line always appears in front of the blue line. Therefore, the phase evolution of E_1_ was indeed abnormal because of its inverse phase velocity, which caused the phase on the detecting surface to vary in an unusual way.

**Figure 4:**
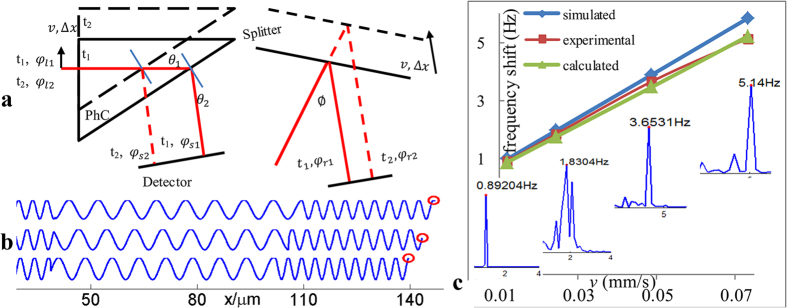
Analysis of the inverse Doppler effect based on the static FDTD method. (**a**) Simplified graph of the two interference branches when the platform moves. (**b**) The spatial field distribution along the signal beam path at different times. The terminal point denoted by the red circle is the detection point. (**c**) Comparison between Δ*f*, Δ*f*_*e*_ and Δ*f*_*c*_. The insets present the frequency spectra of the experimentally measured beat waves.

In the experiment^6^, in which the platform was moving, the optical path changed continuously in both branches of the optical setup, which caused a phase variation on the detecting surface. Those two branches can be simplified as a signal beam with light traveling throughout the LH-PhC and a reference beam with light reflected by the moving splitter. As displayed in Fig. 4(a), at time t_1_, the phase of the light source is *φ*_*l1*_, and the phases of the signal and reference beams on the detecting surface are *φ*_*s1*_ and *φ*_*r1*_, respectively. After the platform moves distance Δ*x* at velocity *v*, at time t_2_, the phases became *φ*_*l2*_, *φ*_*s2*_and *φ*_*r2*_, respectively. During the interval Δ*t* = *t*_*2*_ − *t*_*1*_, the phase increments for each item are *φ*_*s*_ = *φ*_*s2*_ − *φ*_*s1*_ and *φ*_*r*_ = *φ*_*r2*_ − *φ*_*r1*_. From this perspective, the frequency shift can be expressed as Δ*f* = *|φ*_*s*_ − *φ*_*r*_*|*/(*2π *· Δ*t*).

Because the software we used (FDTD solutions) weakly treats moving objects, we have to divide the continuous movement into a series of discrete motions at different times. We assumed that *v* = *0.0244 mm*/*s*, as was one of the speed used in the experiment. As the platform moved away from the detector step by step over Δ*x* (Δ*x* = 1 μm), the initial phases *φ*_*li*_ for each time *t*_*i*_ were adjusted to their appropriate values in the software using the equation (*λ*/*c*)/(*i *· Δ*x*/*v*) = *2π*/*φ*_*li*_. Therefore, by employing the analysis mentioned above, the spatial electrical field distributions of the signal beam for three different moments along optical path are shown in Fig. 4(b), where the red circle indicates the detection point. The phase differences Δ*φ*_*s*_ between each adjacent time can be obtained by subtracting the former value of the detection point from the latter. For reference beam, the change in light path was Δ*l* = *v *· Δ*t* · sin(*ϕ*/2 + *θ*_*1*_ + *θ*_*2*_)/sin(*ϕ*/*2*), which can be inferred from the geometrical relationship shown in Fig. 3(a) by using the sine theorem. Using the same subtraction operation, the phase difference Δ*φ*_*r*_ = *2π *· Δ*l*/*λ* could also be obtained. Consequently, the frequency difference Δ*f* of the two arms was calculated to be 1.949 Hz. Other frequency difference data corresponding to the velocities used in the experiment, namely, 0.0122 mm/s, 0.0244 mm/s and 0.0732 mm/s, were also be obtained, as shown in Fig. 4(c), and the measured frequency shifts Δ*f*_*e*_ and theoretical frequency shifts Δ*f*_*c*_ calculated from the expression are also presented.

The calculated Δ*f* data were unambiguously consistent with the measured Δ*f*_*e*_ data and the theoretical Δ*f*_*c*_ data, with a certain error between Δ*f* and Δ*f*_*c*_ that does not affect the judgment of the inverse Doppler effect. It illustrates that the above analysis of the physical mechanism is correct. Part of the discrepancy can be clarified by the spectra of measured beat waves, which is depicted in the insets of Fig. 4(c). Because they are influenced by the measurement noise, the spectra by no means resemble idealized Dirac functions but are expanded in a small range.

### Doppler shift obtained by the dynamic FDTD method

**Figure 5:**
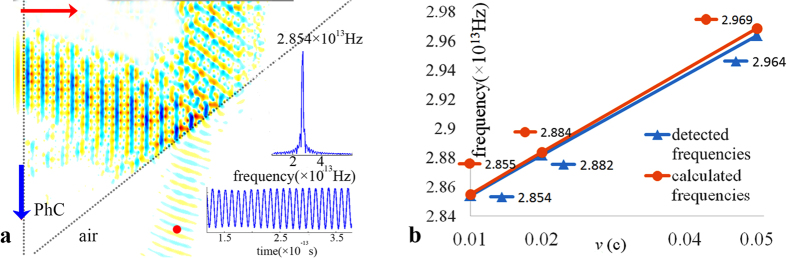
Analysis of the inverse Doppler effect based on the dynamic FDTD method. (**a**) Simulation of light transmitted through the moving PhC at a velocity of 0.05 c. The red dot denotes the position of the detector, the red arrow points in the direction of the emitted light, and the blue arrow points in the direction of which the PhC moves. The inset shows the recorded field obtained at the detection point and its Fourier spectrum. (**b**) Comparison between the detected frequencies *f*_*d*_ and the calculated frequencies *f*_*c*_ at three speeds: 0.01 *c*, 0.02 *c* and 0.05 *c*.

The above analysis was based on the variation of phase change obtained by slicing the continuous motion into a suite of static movements at different time, and the relationship between each movement only serves to add an increased source phase source to the former one. This analysis cannot sufficiently reproduce such a continuous process. In fact, unlike a mechanical wave, the propagation of electromagnetic waves is influenced by the electromagnetic fields at previous times when relative movement occurs because they obey Maxwell’s equations. Specifically, the existing electrical field and magnetic field data from the former time will be added to the new field data at the later time to create a new field distribution, and another field distribution will be produced for the next time using Maxwell’s equations. To this end, we developed an improved dynamic FDTD method. However, this method is limited in handling low-speed motion because the movement at each step time of the FDTD is very small. To ensure appropriate resolution, the grid should be set to a nanometer scale, which would require thousands of hours of simulation time with a general-purpose computer. In this study, after evaluating the time consumption, resolution and analysis requirements, we set the grid size of the FDTD to 0.05 μm, and simulated the inverse Doppler Effect with velocities of 0.01 *c*, 0.02 *c* and 0.05 *c*, in that order. In the simulation, a specified point along the outgoing beam was treated as the detector, at which the electrical field data were recorded at each time. The electrical field distribution for a velocity of 0.05*c* is depicted in Fig. 5(a), the red dot denotes the position of the detector, the red arrow points in the direction of emitted light, and the blue arrow points in the direction in which the PhC moves. Taking the FFT of the record field data from the detector, the detected frequency *f*_*d*_ was found to be 2.854 × 10^13^ Hz. Both the record field and spectrum are shown as insets in Fig. 5(a). Because this frequency shift could not obtain experimentally since such high speeds were not attainable in the experiments, for comparison, a calculated frequency *f*_*c*_ of 2.855 × 10^13^ Hz was obtained by using the expression in^6^, where *f*_*0*_ is the original frequency of the CO_2_ laser source, 2.827 × 10^13^ Hz, and the effective refractive index *n*_*p*_ is −0.5062, which was the same as that in the experiment^6^. Using the same approach, the detected frequencies and calculated frequencies for the remaining two speeds were also obtained, as shown in Fig. 5(b).

The detected values of *f*_*d*_ obtained using the improved dynamic FDTD method at three velocities namely, 0.01 *c*, 0.02 *c* and 0.05 *c* were very close to the calculated values of *f*_*c*_, with a relative error that did not exceeded 5%, which is less than that of the static FDTD method. This finding supports our assumption that the electromagnetic field of the former time affected the later field and influenced the frequency shift when there was relative movement, possibly contributing to the discrepancy mentioned in the discussion of the static FDTD method.

## Discussion

We analyzed the Bloch wave in the PhC by treating it as a summation of a series of spatial harmonics and extracted the main spatial harmonic components. The comparison between the phase changes of the electrical fields in the PhC and those in air identified an unusual component in the PhC, with *v*_*p*_*·k* < *0*, producing a negative refraction phenomenon. Subsequently, in terms of the phase evolution, by using the electrical field data obtained from the static FDTD method, we calculated the frequency difference between the signal and the reference beams at detection point. The result demonstrates that the experimental data were identical to the calculated values obtained with the static FDTD method, which supposes that the component E_1_ that yields to negative refraction leads to the inverse Doppler effect. To address the insufficiency of the static FDTD method for handling moving objects, and to further verify our analysis, we improved on the traditional FDTD method and simulated the inverse Doppler effect with the same PhC parameters used in the experiment. Furthermore, the simulated result generated by the dynamic FDTD method was consistent with the calculated value by using the expression in ref. 6, which strongly supports the rationality of considering the contribution of the LH-PhC by simply using a negative *n*_*p*_. Hence, the phenomenological result of our experiment can be explained by a clearly physical mechanism. Additionally, our study provides a new way to analyze the behavior of EM waves travelling in PhC, particularly, in moving objects.

## Methods

### EFS

The EFS varies with the parameters of the PhC, such as the radius of the rod (hole), refractive index and shape. From the EFS, we can derive the expression *n*_*p*_ = *k*/(*2π* · *ω*). Thus, the relationship between the radius of the rod and the refractive index is known, and we can use it to infer the radius of the rod from the measured refractive index.

### FFT and filter

We extracted the main components of the complex spatial field distribution in the PhC. We then applied the FFT to the original data and identified the desired peak in the spectrum. Specifically, we retained the amplitude of the required frequency, and set other amplitudes to zero and then used the iFFT to reconstruct the required component without losing the phase information.

### Dynamic FDTD method

In contrast to the static FDTD method in which objects remain stationary, for moving objects, the dynamic FDTD method considers the fact that the existing electrical fields and magnetic fields at earlier times should be added to the new field data at later time to create a new field distribution; this new field can then be used to produce another field distribution at the next time according to Maxwell’s equations. In the dynamic FDTD method, at each sliced time, the position of PhC was changed by varying its parameters (namely, the dielectric constant and magnetic permeability) in each grid. The electrical field and magnetic field were stored as the initial field for the next calculation time.

## Additional Information

**How to cite this article**: Jiang, Q. *et al.* Mechanism Analysis of the Inverse Doppler Effect in Two-Dimensional Photonic Crystal based on Phase Evolution. *Sci. Rep.* **6**, 24790; doi: 10.1038/srep24790 (2016).
